# Shared and divergent mental health characteristics of *ADNP*-, *CHD8*- and *DYRK1A*-related neurodevelopmental conditions

**DOI:** 10.1186/s11689-024-09532-1

**Published:** 2024-04-15

**Authors:** Emily Neuhaus, Hannah Rea, Elizabeth Jones, Hannah Benavidez, Conor Miles, Alana Whiting, Margaret Johansson, Curtis Eayrs, Evangeline C. Kurtz-Nelson, Rachel Earl, Raphael A. Bernier, Evan E. Eichler

**Affiliations:** 1 Department of Psychiatry and Behavioral Sciences, University of Washington School of Medicine, Seattle, WA USA; 2 Center On Child Health, Behavior, and Development, Seattle Children’s Research Institute, Seattle, WA USA; 3 Department of Psychology, University of Washington, Seattle, WA USA; 4 Department of Pediatrics, Indiana University School of Medicine, Indianapolis, IN USA; 5 Department of Genome Sciences, University of Washington School of Medicine, Seattle, WA USA; 6 Howard Hughes Medical Institute, Seattle, WA USA

**Keywords:** Neurodevelopmental conditions, Autism, ASD, Phenotyping; genetics, *ADNP*, *CHD8*, *DYRK1A*, Mental health

## Abstract

**Background:**

Neurodevelopmental conditions such as intellectual disability (ID) and autism spectrum disorder (ASD) can stem from a broad array of inherited and de novo genetic differences, with marked physiological and behavioral impacts. We currently know little about the psychiatric phenotypes of rare genetic variants associated with ASD, despite heightened risk of psychiatric concerns in ASD more broadly. Understanding behavioral features of these variants can identify shared versus specific phenotypes across gene groups, facilitate mechanistic models, and provide prognostic insights to inform clinical practice. In this paper, we evaluate behavioral features within three gene groups associated with ID and ASD – *ADNP*, *CHD8*, and *DYRK1A* – with two aims: (1) characterize phenotypes across behavioral domains of anxiety, depression, ADHD, and challenging behavior; and (2) understand whether age and early developmental milestones are associated with later mental health outcomes.

**Methods:**

Phenotypic data were obtained for youth with disruptive variants in *ADNP*, *CHD8*, or *DYRK1A* (*N* = 65, mean age = 8.7 years, 40% female) within a long-running, genetics-first study. Standardized caregiver-report measures of mental health features (anxiety, depression, attention-deficit/hyperactivity, oppositional behavior) and developmental history were extracted and analyzed for effects of gene group, age, and early developmental milestones on mental health features.

**Results:**

Patterns of mental health features varied by group, with anxiety most prominent for *CHD8*, oppositional features overrepresented among *ADNP*, and attentional and depressive features most prominent for *DYRK1A*. For the full sample, age was positively associated with anxiety features, such that elevations in anxiety relative to same-age and same-sex peers may worsen with increasing age. Predictive utility of early developmental milestones was limited, with evidence of early language delays predicting greater difficulties across behavioral domains only for the *CHD8* group.

**Conclusions:**

Despite shared associations with autism and intellectual disability, disruptive variants in *ADNP*, *CHD8*, and *DYRK1A* may yield variable psychiatric phenotypes among children and adolescents. With replication in larger samples over time, efforts such as these may contribute to improved clinical care for affected children and adolescents, allow for earlier identification of emerging mental health difficulties, and promote early intervention to alleviate concerns and improve quality of life.

**Supplementary Information:**

The online version contains supplementary material available at 10.1186/s11689-024-09532-1.

## Background

Neurodevelopmental conditions such as intellectual disability (ID) and autism spectrum disorder (ASD) can stem from a broad array of inherited and de novo genetic differences, including copy number variants (CNVs) and single likely gene-disrupting (LGD) variants [21, 52, 62]. Although they account collectively for approximately 25% of ASD cases, CNVs and LGD variants conferring increased likelihood for ID and ASD are individually rare among research samples [28, 29, 31, 39]. Characterization of the complex phenotypes arising from these sources is thus incomplete, but nuanced understanding of the developmental implications of different genetic influences has tremendous potential to improve clinical care, elucidate mechanisms influencing outcomes, and inform etiological models linking genes to behavior.

Among the many LGD variants influencing neurodevelopmental outcomes, those affecting the genes *ADNP*, *CHD8*, and *DYRK1A* are among the most prevalent and consistently identified within cohorts ascertained for ASD and developmental disabilities [21, 52, 62]. Individuals with disruptions to *ADNP* (a transcription factor-encoding gene involved in chromatin remodeling) exhibit facial dysmorphology, early-emerging gastrointestinal and feeding difficulties, vision and hearing concerns, musculoskeletal differences, and both cardiac and endocrine complications [25, 60]. LGD variants to *CHD8* (a chromatin remodeler associated with regulation of β-catenin and Wnt) result in an overgrowth syndrome that most commonly includes macrocephaly and tall stature, sleep disturbance, gastrointestinal problems, and hypotonia [6, 40, 46, 47]. Clinical presentation of LGD variants to *DYRK1A* (a dual kinase regulating cell proliferation and differentiation) includes microcephaly and distinct facial features, persistent feeding and gastrointestinal difficulties, seizures and hypertonia, short stature, and vision problems [13, 58, 59].

Efforts to characterize brain-based phenotypes of these variants have typically centered on clarifying their relative prevalence of ASD and ID, as well as investigating profiles of ASD features within and across groups. Among *ADNP* patients described in the literature, ID has been nearly universal, with the majority of patients meeting criteria for ASD diagnosis as well [4, 25, 60]. Among those with ASD, features related to unusual social approach, lack of interest in peers, sensory-seeking behaviors, and repetitive motor movements are prominent in standardized evaluations, along with relative strengths in nonverbal communication [4, 27, 56, 61]. Among those with *CHD8* events, a somewhat different phenotype emerges, with ID that is less severe on average [3], but very high rates of ASD diagnosis among individuals who are rigorously evaluated with standardized measures [6, 11, 62]. When compared to individuals with LGD variants in other genes, those with *CHD8* variants may exhibit decreased social motivation and more marked repetitive/restricted behaviors [5], as well as heightened auditory sensitivities [27]. *DYRK1A* is again associated with ID (often moderate to severe) in nearly 90% of patients [13, 32], along with frequent language delays and motor speech disorders [41]. Comprehensive standardized assessment of the behavioral phenotype yields diagnostic rates of 85% for ASD among individuals with LGD variants to *DYRK1A* [32], and quantitative assessments of ASD features suggest relative strengths in social motivation [41], but more marked impact on repetitive and sensory-oriented behaviors [27, 32, 41].

An essential component in characterizing the behavioral phenotype of these conditions is to consider the presence of psychiatric features within specific gene groups. Regardless of etiology, individuals with ASD more broadly exhibit elevated rates of mental health diagnoses and subclinical symptoms relative to the general population [26, 33, 45], and genetic conditions associated with ASD may thus carry a similar concern. According to population-derived estimates, approximately 70% of children and adolescents with ASD have at least one mental health condition, with 40% experiencing multiple conditions [55]. Precise prevalence rates of specific diagnoses vary across estimates and shift over the course of development, but anxiety, depression, and attention-deficit/hyperactivity disorder (ADHD) appear to be the most common psychiatric diagnoses among individuals with ASD, with notable rates of disruptive behavior disorder as well [33, 34].

Existing literature on the psychiatric phenotypes of LGD variants lags behind our understanding of ASD and ID in these populations, but evidence emerging from studies of these variants individually documents overlapping areas of concern. For example, *ADNP* has been associated with marked anxiety and obsessive–compulsive behavior, as well as externalizing features such as aggressive behavior, temper tantrums, and ADHD [23, 60, 61]. Similarly, individuals with LGD variants to *CHD8* display elevated rates of anxiety disorders and ADHD diagnoses, and related traits (e.g., hyperactivity) have been documented among individuals who did not have formal diagnoses of ADHD [12, 23]. Tantrums, aggression, and self-injurious behaviors have been noted as well in individuals with LGD variants to CHD8 [23, 40]. Among children with *DYRK1A* syndrome, concerns for withdrawal, attention problems, and depressive symptoms appear to be most prevalent, with relatively fewer concerns related to anxiety, aggressive, or oppositional behavioral [18]. Across *DYRK1A* samples with broader age ranges, formal diagnoses and subthreshold features of hyperactivity and anxiety have been reported [13, 23, 58].

From this emerging literature, we can observe areas of shared behavioral features as well as potential points of divergence between gene groups. However, direct group comparisons are infrequent, as *ADNP*-, *CHD8*-, and *DYRK1A*-associated conditions are individually rare in the general population and have been identified relatively recently. In addition, ascertainment and assessment procedures vary across studies, affecting both prevalence rates for mental health diagnoses and our ability to contrast findings across different research groups. Thus, the extent to which psychiatric phenotypes differ across these groups remains unclear. In this paper, we pursue two related aims. First, we aim to describe multiple domains of mental health features observed among youth with rare LGD variants in the genes described above – *ADNP*, *CHD8*, and *DYRK1A* – with the goal of understanding shared and divergent features. By following a ‘genetics first’ approach (in which recruitment is defined by *genetic*, rather than *behavioral*, diagnosis; [30, 57] and applying a standardized assessment protocol, we are able to evaluate mental health profiles in a more systematic and cohesive manner.

Second, we aim to explore developmental patterns in mental health features for these groups, through consideration of both chronological age and early developmental milestones. Because of the rarity and relatively recent recognition of these genetic conditions, prospective longitudinal research charting trajectories of development over time is incomplete [7]. In its absence, one method to assess change over time is through exploration of developmental milestones, particularly those related to motor and communication development, which are compelling for several reasons. First, delays in early motor and language development may predict internalizing difficulties later in childhood among the general population [24, 54], suggesting they may have predictive validity for mental health concerns among youth with neurodevelopmental conditions as well. Second, delays in milestones are more common among individuals with ASD-associated genetic differences relative to those with ASD without an identified genetic cause, and may be most severe among those with single-gene conditions such as those included here [64]. Third, early milestones are routinely monitored in medical settings and may be quantified easily and noninvasively, and so could be readily leveraged to inform clinical care for affected youth. As such, the timing of developmental milestones and their association with later outcomes may be both relevant to our scientific understanding of neurodevelopmental outcomes and clinically meaningful for youth with LGD variants to the genes explored here.

No study to date has examined how developmental milestones might be associated with psychiatric features for youth with *ADNP*, *CHD8*, and *DYRK1A* variants, but findings from our research group indicate that motor and language milestones offer predictive insight into neurodevelopment more broadly [3]. Moreover, that work suggests differential predictive patterns across different genetic groups, such that age of independent walking predicts later cognitive outcomes among individuals with disruptive variants to *ADNP*, whereas language milestones such as age of acquisition of single words and phrases may predict cognitive and adaptive outcomes for those with LGD variants to *CHD8* and *DYRK1A* [3].

Together, these emerging findings suggest that timing of developmental milestones may carry potential to distinguish more specific developmental courses across these gene groups, implicating underlying neurobiological systems and yielding predictive clinical implications for medical and educational supports [3, 64]. In this paper, we aim to extend these efforts by exploring the role of developmental milestones in understanding mental health. By drawing on extensive phenotypic data collected using standardized research procedures, we seek to identify points of similarity and divergence across *ADNP*, *CHD8*, and *DYRK1A* neurodevelopmental conditions to better inform clinical practice and mechanistic models.

## Methods

### Participants

Participants were enrolled in a long-running ‘genetics first’ study at the University of Washington (R01MH101221), through which individuals with rare variants in ASD-associated genes completed in-depth phenotyping procedures [5, 32]. For the current manuscript, participants with LGD variants in *ADNP*, *CHD8*, or *DYRK1A* were included, with variants confirmed through targeted sequencing or exome sequencing through a research study, or through review of clinical genetic testing lab reports. This yielded a sample of 65 individuals. Full genetic characterization is presented in Supplementary Materials, Table S1.

All study procedures were conducted in accordance with the University of Washington Institutional Review Board. Participants and their legal guardians provided informed assent, consent, and permission as appropriate. Following enrollment, research clinicians conducted comprehensive clinical and behavioral evaluations, often via onsite family visits to the University of Washington with a minority via home visit or telehealth. Assessments included cognitive assessment, caregiver interview, and extensive caregiver questionnaires. Diagnostic testing for ASD was overseen by licensed psychologists, using research reliable administrations of the Autism Diagnostic Observation Schedule (ADOS-2) [35] and Autism Diagnostic Interview, Revised (ADI-R) [36, 37].

Table 1 presents demographic and phenotypic characteristics for the sample as a whole and separately by gene group. Note that IQ data were available for only a subset of participants (approximately 70% of the sample), as cognitive testing was completed only for those evaluated onsite or through home visit (not telehealth). Gene groups did not differ significantly in verbal IQ, *F*(2,43) = 1.10, *p* = 0.34, or in nonverbal IQ, *F*(2,44) = 1.28, *p* = 0.29, for those with available IQ data.

**Table 1:** Means, standard deviations, and ranges for participant demographic and phenotypic characteristics

	***Full Sample*** ***N* = *65***	***ADNP*** ***N* = *21***	***CHD8*** ***N* = *18***	***DYRK1A*** ***N* = *26***
*Age in years*	8.7 (4.3)	7.5 (3.3)	9.4 (3.8)	9.1 (5.2)
	Range: 3.9 to 18.7	Range: 3.9 to 15.6	Range: 5.4 to 16.8	Range: 4.0 to 18.7
*Sex Assigned at Birth*	26 female	9 female	5 female	12 female
	39 male	12 male	13 male	14 male
*Verbal IQ* *N* = 46	49.1 (27.8)	48.6 (23.7)	60.2 (32.0)	44.7 (27.8)
	Range: 4 to 119	Range: 16 to 97	Range: 15 to 101	Range: 4 to 119
*Nonverbal IQ* *N* = 47	50.2 (26.3)	46.0 (24.1)	61.2 (27.1)	47.3 (26.6)
	Range: 12 to 133	Range: 16 to 99	Range: 20 to 100	Range: 12 to 133
*% Autism Spectrum Disorder*	Yes: 47 (72.3%)	Yes: 9 (42.9%)	Yes: 16 (88.9%)	Yes: 22 (84.6%)
	No: 12 (18.5%)	No: 9 (42.9%)	No: 0	No: 3 (11.5%)
	Unsure: 6 (9.2%)	Unsure: 3 (14.3%)	Unsure: 2 (11.1%)	Unsure: 1 (3.9%)
*CBCL Anxiety Problems*	60.4 (10.6)	60.9 (11.1)	64.6 (9.9)	57.1 (9.8)
	Range: 50 to 82	Range: 50 to 79	Range: 50 to 82	Range: 50 to 79
*CBCL Depressive Problems*	63.2 (9.1)	63.1 (9.2)	66.4 (8.7)	61.1 (9.0)
	Range: 50 to 82	Range: 50 to 82	Range: 52 to 82	Range: 50 to 79
*CBCL ADHD*	63.0 (7.9)	64.6 (8.2)	61.4 (7.7)	62.8 (7.9)
	Range: 50 to 80	Range: 51 to 78	Range: 50 to 80	Range: 50 to 76
*CBCL Oppositional*	57.5 (8.6)	61.9 (9.8)	56.4 (7.3)	54.7 (7.2)
	Range: 50 to 77	Range: 50 to 77	Range: 50 to 71	Range: 50 to 73

Verbal IQ available for 46 participants. Nonverbal IQ available for 47 participants. *CBCL* Child Behavior Checklist [1]. CBCL domains are reported in T-scores with a mean of 50 and standard deviation of 10

### Measures

#### Cognitive ability

Procedures for cognitive assessment were modeled on those of the Simons Simplex Collection [19]. Verbal and nonverbal IQ were measured with the Differential Ability Scales, 2nd Edition (DAS-II) [15], Wechsler Abbreviated Scale of Intelligence, 2nd Edition (WASI-2) [63], or Mullen Scales of Early Learning [42], with test selection based on age and ability level. Verbal IQ was composed of the Verbal Composite score of the DAS-II, Verbal Comprehension score of the WASI-2, or sum of verbal subdomains (Receptive Language, Expressive Language) on the Mullen. Nonverbal IQ was composed of the Special Nonverbal Composite Score on the DAS-II, Perceptual Reasoning score of the WASI-2, or sum of the nonverbal domains (Visual Reception, Fine Motor) of the Mullen. When valid, standardized IQ scores with a mean of 100 (SD of 15) were extracted. When a participant’s performance was below the floor of a measure or a measure was administered out of age level, ratio IQ scores (100 × mental age equivalent / chronological age) were computed instead.

#### Developmental milestones

Information about milestones was gathered through structured interviewing with the Autism Diagnostic Interview – Revised (ADI-R) [36, 37]. Responses to Item 5 (First Walked Unaided) and Item 9 (First Single Words) were extracted, reflecting acquisition of independent walking and acquisition of meaningful single words, respectively. Independent walking was coded as “no delay” if it was achieved by 18 months of age, and coded as “delay” if achieved later or not at all. Similarly, acquisition of single words was coded as “no delay” if it was achieved by 24 months of age, and coded as “delay” if achieved later or not at all. The decision to dichotomize responses in this way was based on two factors. First, a subset of participants had not met these milestones and so did not have a true “age of acquisition” at the time of study participation. Second, the ADI-R allows categorical coding of caregivers’ responses when needed (e.g., “[age of acquisition] not known but apparently delayed”). As a result, continuous data were not available for all participants, whereas a dichotomous approach allowed us to retain all participants with ADI-R data.

#### Behavioral features

Mental health features were assessed through caregiver report using the age-appropriate version of the Child Behavior Checklist (CBCL) [1], a well-established questionnaire that assesses a broad range of behavioral and mental health domains. For each item on the CBCL, caregivers are asked to indicate the extent to which it is “Not True”, “Somewhat or Sometimes True”, or “Very True or Often True” of their child. Standardized scores (normed for age and sex) can then be derived for a number of clinical domains. Resulting T-scores have a mean of 50 (standard deviation of 10) in normative samples [1], with scores between 65 to 69 constituting the borderline clinical range and scores exceeding 69 constituting the clinical range. For the current analyses, we extracted standardized scores for the following DSM-5-oriented domains: Anxiety Problems (e.g., fear, worry, self-consciousness), Depressive Problems (e.g., sadness, guilt, fatigue), Attention Deficit/Hyperactivity Problems (e.g., impulsivity, inattention), and Oppositional Defiant Problems (e.g., arguing, disobedience, tantrums). Domains were selected due to their comparability across the two versions of the CBCL appropriate for the sample age range, which allowed for consistent outcome variables across the full sample. Both continuous T-scores as well as categorical ranges (average versus elevated above 65) were used.

### Analytic approach

Analysis followed two approaches. For our first aim, characterizing shared and divergent features across our three gene groups, we computed a 3 × 4 ANOVA with gene group (*ADNP*, *CHD8*, *DYRK1A*) as a between-subjects factor and behavioral domain (anxiety, depressive problems, ADHD, oppositional) as a within-subjects factor. Interaction effects were prioritized over main effects during interpretation, consistent with recommendations [48], and significant effects were further analyzed with ANOVAs. Outcomes in these models were CBCL T-scores and thus were continuous measures. Next, to understand whether gene group membership was associated with elevated levels of behavioral features defined categorically, we computed chi-square analyses to compare group distributions across average versus elevated score ranges defined dichotomously as below or above T-scores of 65, respectively.

For our second aim, understanding developmental patterns in mental health features, we first computed correlations between chronological age and continuous CBCL T-scores. Because T-scores on the CBCL are sex- and age-normed, significant correlations represent divergence from normative trajectories over the course of development, whereas nonsignificant correlations represent maintenance of a consistent position relative to peers. To investigate potential predictive effects of early developmental milestones, we then computed an ANCOVA with CBCL T-scores as outcomes, and absence/presence of delay in age of walking and absence/presence of delay in age at single word acquisition as between-subjects factors. Age at assessment was entered as a continuous covariate. Models were run separately for each gene group to understand developmental patterns unique to each group.

## Results

### Aim 1: Shared and divergent behavioral features

**Fig. 1:**
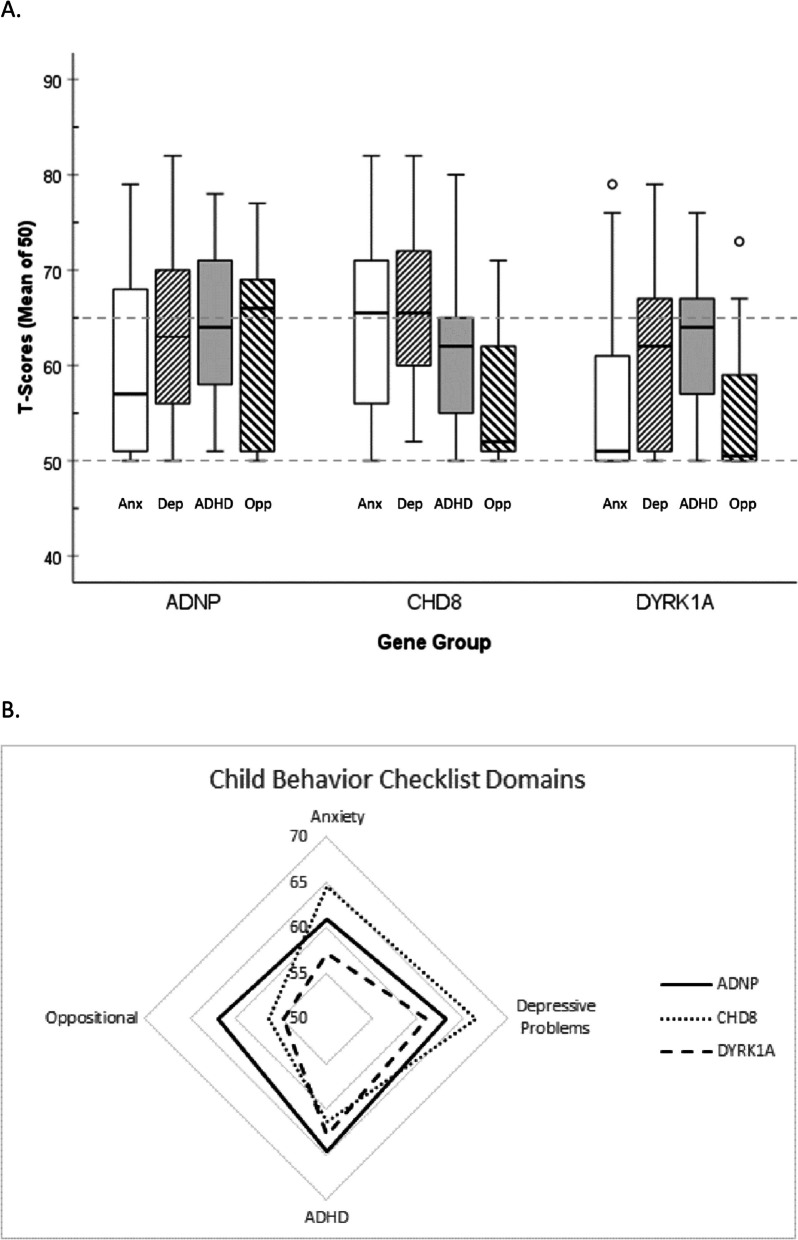
Distributions of T-scores for four domains of the Child Behavior Checklist by gene group. Notes: In panel A, gray horizontal lines at T-scores of 50 and 65 indicate population mean (T = 50) and clinical threshold (T = 65), respectively. T-scores have a mean of 50 and SD of 10. CBCL, Child Behavior Checklist [1]

Analyses revealed a significant main effect of behavioral domain on CBCL scores, *F*(3, 186) = 9.21, *p* < 0.001, partial eta^2^ = 0.129. However, this was qualified by a significant Domain x Gene interaction effect, *F*(6, 186) = 2.95, *p* = 0.009, partial eta^2^ = 0.087, indicating different profiles of behavioral features across the gene groups (see Table 1 and Fig. 1). Although IQ data were not available for the full sample, this omnibus model was then tested with verbal IQ and nonverbal IQ entered as covariates to account for potential differences in verbal or nonverbal IQ scores across the groups. Because the pattern of results was largely unchanged (Domain x Gene interaction *F*(6, 123) = 2.98, *p* = 0.009, partial eta^2^ = 0.127; main effect of CBCL domain *F*(3, 123) = 6.77, *p* < 0.001, partial eta^2^ = 0.142; main effect of gene group *F*(2, 41) = 2.94, *p* = 0.064, partial eta^2^ = 0.125), subsequent analyses omitted IQ covariates in order to include the full sample and maintain parsimonious models.

Within-subjects ANOVAs were then computed for each gene group individually to understand the significant Domain x Gene interaction. Simple contrasts were computed, in which scores for each domain were compared to that of the last domain (oppositional behavior). For the *ADNP* group, the effect of CBCL domain was not significant, Greenhouse–Geisser *F*(3, 60) = 0.87, *p* = 0.46, partial eta^2^ = 0.042, indicating relatively uniform scores across the CBCL domains for participants in the *ADNP* group. In contrast, within the *CHD8* group, the effect of domain was significant, *F*(3, 51) = 7.521, *p* < 0.001, partial eta^2^ = 0.307, with contrasts revealing significant elevations in anxiety, *F*(1, 17) = 18.18, *p* < 0.001, partial eta^2^ = 0.517, depressive problems, *F*(1, 17) = 23.43, *p* < 0.001, partial eta^2^ = 0.580, and ADHD features, *F*(1, 17) = 5.23, *p* = 0.035, partial eta^2^ = 0.235, relative to oppositional behavior. The effect of CBCL domain was also significant for the *DYRK1A* group, *F*(3, 75) = 9.14, *p* < 0.001, partial eta^2^ = 0.268, and was such that scores for depressive problems, *F*(1, 25) = 15.04, *p* < 0.001, partial eta^2^ = 0.376, and scores for ADHD, *F*(1, 25) = 21.14, *p* < 0.001, partial eta^2^ = 0.458, were significantly higher than those for oppositional behavior.

**Fig. 2:**
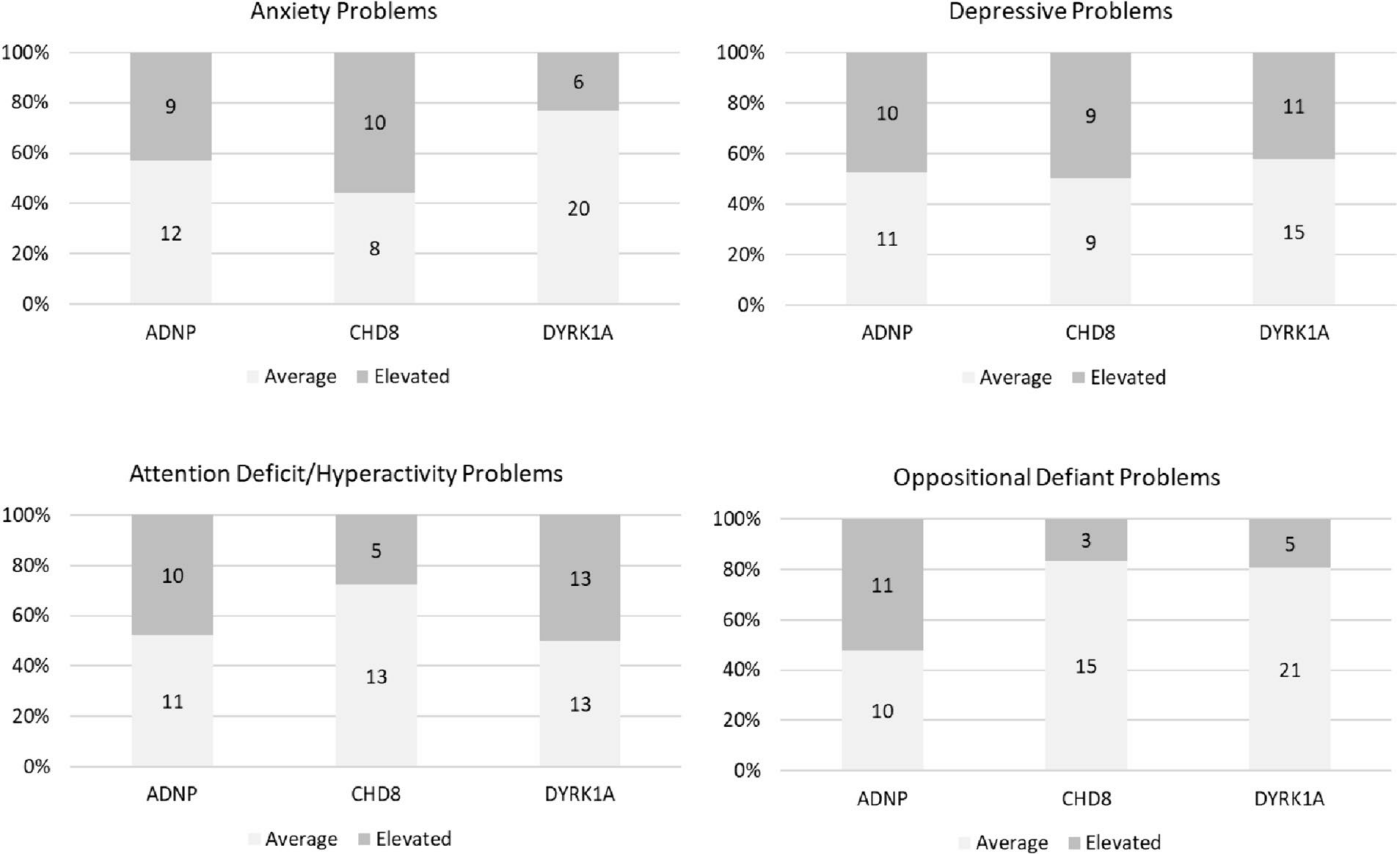
Proportions of participants in each gene group with Child Behavior Checklist T-scores in the average and elevated (at or above 65) ranges

Next, we aimed to compare gene groups with respect to clinical severity of behavioral features. Figure 2 presents group distributions of behavioral features, indicating the percentage of each gene group with average versus elevated scores on each domain. With regard to anxiety, an omnibus *χ*^*2*^ test comparing the observed group distributions indicated a trend-level effect, *χ*^*2*^(2) = 4.99, *p* = 0.082, such that the *DYRK1A* group was less likely to have elevated anxiety (adj. std. residual = -2.1) whereas the *CHD8* group may be overrepresented among those with elevated anxiety (adj. std. residual = 1.8). As these effects did not meet conventional significance thresholds, they should be considered with caution. Results for oppositional features did meet significance, *χ*^*2*^(2) = 8.07, *p* = 0.018, and were such that the *ADNP* group was overrepresented among those with elevated levels of oppositional behavior, (adj. std. residual = 2.8). Finally, models for depressive problems, *χ*^*2*^(2) = 0.25, *p* = 0.869, and ADHD,* χ*^*2*^(2) = 2.40, *p* = 0.301, were not significant with regard to group distribution.

### Aim 2: Developmental patterns

**Fig. 3:**
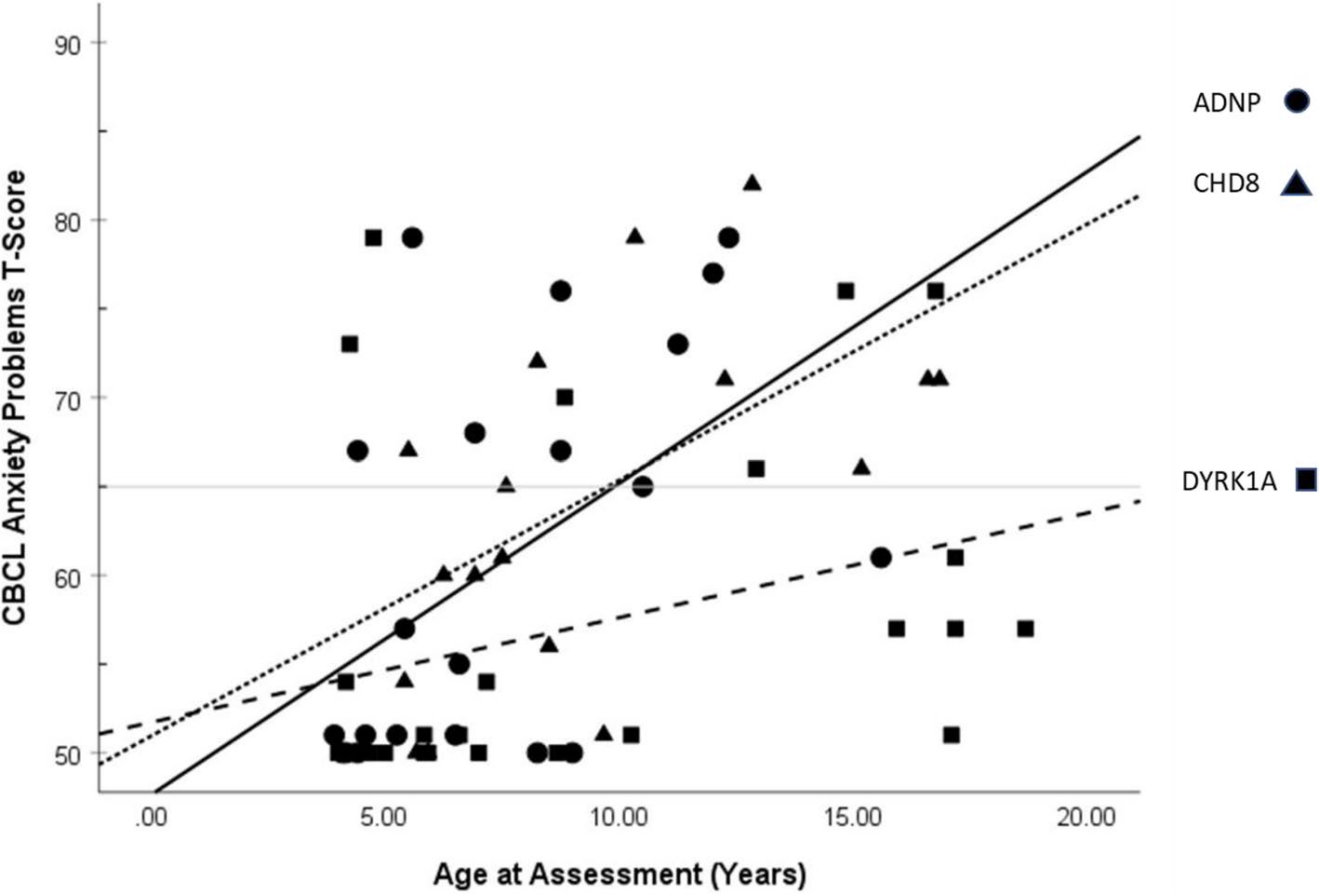
Scatterplot showing correlations between participant age at assessment and Child Behavior Checklist Anxiety Problems T-Scores for each gene group. Notes: Gray horizontal line at T-score of 65 indicates clinical threshold. T-scores have a mean of 50 and SD of 10. CBCL, Child Behavior Checklist [1]

Correlations between CBCL T-scores and participant age are presented in Table 2. For the full sample, anxiety T-scores were positively correlated with age, *r* = 0.394, *p* = 0.001, 95% CI [0.17, 0.58], such that higher scores were associated with older age. Because T-scores are age- and sex-normed, a positive correlation indicates increasing divergence (elevation, in this case) relative to typical developmental fluctuations over time. When considering each gene group individually, this positive association held for both the *ADNP*, *r* = 0.52, *p* = 0.016, 95% CI [0.11, 0.78], and *CHD8* groups, *r* = 0.55, *p* = 0.018, 95% CI [0.11, 0.81]. See Fig. 3. This effect appeared to be unique to anxiety, as there were no significant associations between age and CBCL T-score for depressive problems, ADHD, or oppositional behavior, *p*s > 0.39.

**Table 2:** Correlations between age and CBCL T-scores for the full sample and by gene group

	***Anxiety***	***Depressive***	***ADHD***	***Oppositional***
*Full Sample* *N* = *65*	*r* = .39	*r* = .11	*r* = .04	*r* = -.09
	*p* = .001	*p* = .39	*p* = .75	*p* = .49
	95% CI [.17, .58]	95% CI [-.14, .34]	95% CI [-.21, .28]	95% CI [-.33, .16]
*ADNP group* *N* = *21*	*r* = .52	*r* = -.01	*r* = .18	*r* = .13
	*p* = .016	*p* = .95	*p* = .45	*p* = .59
	95% CI [.11, .78]	95% CI [-.44, .42]	95% CI [-.28, .56]	95% CI [-.32, .53]
*CHD8 group* *N* = *18*	*r* = .55	*r* = .13	*r* = .05	*r* = -.08
	*p* = .018	*p* = .61	*p* = .85	*p* = .76
	95% CI [.11, .81]	95% CI [-.36, .56]	95% CI [-.43, .51]	95% CI [-.52, .41]
*DYRK1A group* *N* = *26*	*r* = .32	*r* = .16	*r* = .03	*r* = -.11
	*p* = .12	*p* = .45	*p* = .88	*p* = .59
	95% CI [-.08, .63]	95% CI [-.25, .51]	95% CI [-.36, .41]	95% CI [-.48, .29]

*CBCL* Child Behavior Checklist [1]

Models investigating developmental milestones were examined next. See Table 3 for developmental characteristics by group.

**Table 3:** Rates of delay and mean age of acquisition for developmental milestones

	***Full Sample***	***ADNP***	***CHD8***	***DYRK1A***
***Independent Walking***	***N* = 64**	***N* = 20**	***N* = 18**	***N* = 26**
*No delay (Met by 18 mos)*	23 (35.9%)	4 (20.0%)	8 (44.4%)	11 (42.3%)
*Delayed (Not met by 18 mos)*	41 (64.1%)	16 (80.0%)	10 (55.6%)	15 (57.7%)
*Age (months) if achieved (Mean, SD)*	22.8 (8.2)	25.5 (8.2)	19.6 (5.8)	23.0 (9.2)
	Range: 12 to 54	Range: 17 to 48	Range: 12 to 36	Range: 12 to 54
***Single Word Acquisition***	***N* = 63**	***N* = 20**	***N* = 17**	***N* = 26**
*No delay (Met by 24 mos)*	16 (25.4%)	3 (15.0%)	8 (47.1%)	5 (19.2%)
*Delayed (Not met by 24 mos)*	47 (74.6%)	17 (85.0%)	9 (52.9%)	21 (80.8%)
*Age (months) if achieved (Mean, SD)*	37.0 (23.0)	39.0 (14.4)	29.2 (24.2)	42.2 (26.9)
	Range: 9 to 120	Range: 18 to 72	Range: 9 to 108	Range: 11 to 120

Within the *ADNP* group, the main effect of CBCL domain was marginally significant, Greenhouse–Geisser *F*(2.17, 34.76) = 3.38, *p* = 0.071, partial eta^2^ = 0.14, and there was a significant Domain x Age interaction effect, Greenhouse–Geisser *F*(2.17, 34.76) = 4.28, *p* = 0.019, partial eta^2^ = 0.21, consistent with the positive correlation between age and anxiety problems described above. In addition, the interaction of Domain x Walking Delay was marginal, Greenhouse–Geisser *F*(2.17, 34.76) = 2.72, *p* = 0.076, partial eta^2^ = 0.145; that effect suggested higher scores on depressive problems for participants with a history of delay in walking, *F*(1, 18) = 3.52, *p* = 0.077, partial eta^2^ = 0.16, but should be considered cautiously as it did not meet conventional thresholds for significance. There were no main or interactive effects observed for delays in single word acquisition, *p*s > 0.10.

Within the *CHD8* group, the Domain x Age interaction was marginally significant, with a positive association between anxiety problems and age, Greenhouse–Geisser *F*(2.21, 28.69) = 3.11, *p* = 0.055, partial eta^2^ = 0.19. There was also a significant main effect of delay in single word acquisition, *F*(1, 13) = 4.90, *p* = 0.045, partial eta^2^ = 0.27; participants with a history of delay (acquisition of single words later than 24 months of age) had higher CBCL scores overall relative to participants who acquired single words by 24 months of age. There were no main or interactive effects of delays in walking.

Finally, among the *DYRK1A* group, results again indicated a main effect of CBCL domain (consistent with that described earlier), *F*(3, 66) = 3.12, *p* = 0.032, partial eta^2^ = 0.12, with no main or interactive effects for acquisition of single words, independent walking, or participant age.

## Discussion

This study is among the first to examine mental health features across multiple groups of individuals with disruptive variants in ASD-associated genes. Our data suggest that *ADNP*, *CHD8*, and *DYRK1A* groups may have separable and distinct phenotypes with regard to mental health, despite their shared associations with autism and intellectual disability. Our findings also suggest that differences persist even when accounting for verbal and nonverbal intellectual ability, an important fact when comparing overlapping phenotypes.

### Shared and divergent features

Among youth with LGD variants to *ADNP*, scores for the four behavioral domains measured showed variability within the group, but group means fell generally in the normal range as shown in Fig. 1. We did not find particular elevations among any of the domains when examined within the *ADNP* group. However, when distributions of normal versus elevated scores were examined across groups, we did find that the *ADNP* group was significantly overrepresented among youth with clinically relevant oppositional difficulties. Thus, oppositional behavior may be the area most likely to warrant clinical support for this group. Indeed, emerging qualitative evidence from families of youth affected by *ADNP* variants highlights aggression and associated behaviors as both highly prevalent (endorsed by all families included) and among the most pressing stressors for caregivers [17]. Supports to address challenging behaviors may include medication as well as behavioral interventions that can elucidate the triggers and functions of challenging behaviors, but should also include speech/language therapy to reduce communication barriers that can contribute to externalizing behaviors [17, 44, 53].

Within the *CHD8* group, a contrasting pattern emerged in which oppositional features were significantly lower than anxiety, depression, and ADHD, all of which had group means approaching clinical thresholds. When considered categorically, the *CHD8* group displayed a marginally significant trend toward overrepresentation among those with clinical levels of anxiety symptoms. Taken together, this pattern suggests that internalizing symptoms may be most prominent for these youth. Furthermore, post hoc analyses of medication use in this sample indicate that those with *CHD8* variants were marginally more likely to be using antidepressant medication at the time of study participation (endorsed for approximately 19% of youth) than were our other groups (5% of *ADNP* group, 0% in *DYRK1A* group), *χ*^*2*^(2) = 5.99, *p* = 0.050. To the extent that medication was decreasing symptoms of anxiety and depression for the *CHD8* group, symptom levels reported here may in fact underestimate the prevalence of internalizing difficulties. Of note, features of ADHD were also elevated in this group, consistent with previous reports indicating ADHD among approximately one half of patients with *CHD8* [40]. Together, this reinforces routine screening for both internalizing and externalizing symptoms as an important component to ongoing care for youth with LGD variants to *CHD8*, as many patients would likely benefit from medical and/or behavioral supports within each of these broad domains.

Youth with LGD variants to *DYRK1A* displayed yet a different profile of mental health features, with significant elevations within the domains of depressive problems and ADHD. Compared to the *ADNP* and *CHD8* groups, the *DYRK1A* group was marginally less likely to have elevated levels of anxiety, and group means for both anxiety and oppositional behavior were well within the normal range on the CBCL. While ADHD has been documented previously for individuals with *DYRK1A* syndrome [59] and our findings are consistent in that regard, depressive features have not been identified within the phenotype. As such, this area warrants more study in this population to determine whether concerns are truly elevated or whether findings here reflect overlap between depressive symptoms and somatic effects of *DYRK1A* variants. For example, several items within the Depressive Problems subscale of the CBCL address sleep concerns, including tiredness, lack of energy, and sleeping too little or too much [1]. While this corresponds well with DSM-5 diagnostic criteria for depression (e.g., presence of fatigue, sleep disturbance) [2], these items also overlap with documented physiological features of *DYRK1A* syndrome [14]. As a result, primary sleep disturbances may inflate estimates of depressive features, and it will be important to understand the extent to which core depressive symptoms of lowered mood and anhedonia might be affected in *DYRK1A* moving forward.

Shared across the sample as a whole was a significant positive correlation between age and anxiety, indicating a marked increase in anxiety with increasing age. Because anxiety was assessed with a sex- and age-normed T-score, this positive correlation indicates that youth in our sample are increasingly discrepant from their peers as time goes on. When considered separately, this effect held for both the *ADNP* and *CHD8* groups. It is important to note that our data are cross-sectional in nature and longitudinal data will be needed to confirm these patterns. Nevertheless, our findings imply the need for intentional screening for anxiety so that appropriate supports can be offered. Within the ASD population more broadly, cognitive-behavioral approaches to reduce anxiety can be highly effective (e.g., Facing Your Fears; [49, 49, 50, 50], and adaptations for youth with co-occurring ASD and intellectual disability may be well suited for youth with genetic conditions conferring higher rates of ID [8].

More variable across the groups considered here was the role of early developmental milestones in understanding longer term outcomes. The strongest effect was observed for our *CHD8* group, for whom a history of delay in single word acquisition was significantly associated with higher CBCL scores across the domains assessed. Thus, early language delay may predict heightened mental health concerns later in childhood and adolescence specifically within the CHD8 population. We also observed a trend-level effect related to motor milestones in our *ADNP* group, such that depressive scores were marginally higher among those who had a history of delay in independent walking. These findings extend previous patterns documented by our research group with a partially overlapping participant sample [3], in which language milestones predicted intellectual and adaptive outcomes among individuals with variants in *CHD8*, but age of walking played a unique role in the prediction of verbal and nonverbal IQ scores among individuals with ADNP. Although these groups are characterized by variability in early development and in later outcomes, increasing evidence supports the potential predictive power of early developmental markers in understanding a broad range of intellectual, adaptive, and psychiatric outcomes.

### Limitations and future directions

A primary limitation of this work relates to measurement and quantification of developmental milestones. As described earlier, we chose to dichotomize acquisition of single words and of independent walking, an approach that increased inclusion of participants (including those who had not attained those milestones) yet decreased both statistical power and specificity in age of milestone attainment. Data also consisted of retrospective reports rather than information collected through prospective and/or observational means. Although parents are likely accurate in recalling concrete milestones such as first words or first steps at the level of specificity assessed here [38], a broader range of motor, communication, and social milestones (e.g., sitting, crawling, rate of word acquisition) evaluated in real-time with more precision would be ideal. Such research would also ideally include assessment of the many other genetic, psychosocial, and environmental factors that may predict mental health outcomes later in development.

Similarly, psychiatric features described in the current study were evaluated through a very well-established standardized questionnaire [1], but mental health assessments are rarely developed or extensively validated among individuals with intellectual disability, for whom symptom presentation, communication methods, and/or opportunities for subjective report of internal experiences may differ from standardization samples [10, 20, 22]. Validation of such questionnaires specifically within groups with genetic conditions is particularly incomplete [43]. Evidence from young children with a range of neurogenetic conditions suggests that the factor structure of some symptom scales may differ from that of validation and standardization samples [43], raising the need for validation within more specific groups. Validation of symptom measures that include somatic features may be especially important; as we note earlier with regard to *DYRK1A*, symptoms conventionally attributed to psychiatric concerns (e.g., sleep disturbance with regard to depression, muscle tension or gastrointestinal concerns with regard to anxiety) may overlap with the complex medical phenotypes observed in these conditions. Indeed, psychometric analyses of the CBCL among youth with Down Syndrome (which also has many physiological components) suggest that subscales with physical symptoms (Somatic Problems, Affective/Depressive Problems) may have lower internal consistency than subscales with fewer physical symptoms (Anxiety, ADHD, and Oppositional concerns) [16]. As a result, scores on the CBCL and other measures developed in the general population may be elevated due to multiple, confounded factors, and psychiatric features may be best assessed by using tools that account for these factors, by interpreting standardized measures within the context of an individual’s broader neurodevelopmental history, and by complementing questionnaires with diagnostic interviewing that parse symptoms more specifically.

Finally, as is often true, results presented here are influenced by the methods through which participants were ascertained. More severely affected individuals may be more likely to receive genetic testing through medical settings and, consequently, our phenotypic understanding may not accurately represent individuals who are similar genetically yet more subtly affected phenotypically [3]. Family and cultural preferences likely affect interest in pursuing genetic testing, and financial and insurance barriers limit access for some families who may be interested [9, 51, 65]. Increased availability of screenings for a broader array of genetic conditions could allay some systemic issues related to access and could allow very early identification that would more accurately represent the full phenotypic spectrum. Larger and more representative participant groups would also permit exploration of individual differences (e.g., intellectual ability, sex assigned at birth) that may moderate mental health outcomes, identify psychiatric concerns across a larger developmental span (e.g., emergence of psychosis or other adult-onset conditions), and facilitate discovery of associations between more specific variant characteristics and phenotypic outcome [61].

## Conclusions

Despite shared associations with autism and intellectual disability, data presented here suggest *ADNP*, *CHD8*, and *DYRK1A* may yield variable psychiatric phenotypes among affected children and adolescents, including differential associations with early development. Replication will be important as participant groups grow in size, phenotypic heterogeneity, and demographic diversity, and future efforts may enhance etiological models of neurodevelopmental conditions and shed light on shared and divergent influences on brain structure and function to more fully trace mechanistic pathways from genes to brain to behavior. For affected families, efforts may contribute to improved clinical care for children and adolescents, allowing for earlier identification of emerging mental health difficulties and more proactive intervention to alleviate those concerns and improve quality of life.

### Supplementary Information

**Additional file 1: Table S1.** Genetic characterization of the included sample.

## Data Availability

The datasets generated and analyzed in the current study are available in part through the National Database for Autism Research (NDAR) study #2093. The remaining data are available from the corresponding author upon reasonable request.

## Abbreviations

ADHD: Attention-deficit/hyperactivity disorder
ADI-R: Autism Diagnostic Interview, Revised
ADOS-2: Autism Diagnostic Observation Schedule, 2nd Edition
ASD: Autism spectrum disorder
CBCL: Child Behavior Checklist
CNV: Copy number variants
DAS-2: Differential Ability Scales, 2nd Edition
ID: Intellectual disability
LGD: Likely gene-disrupting
WASI-2: Wechsler Abbreviated Scale of Intelligence, 2nd Edition

## References

[1] Achenbach T, Rescorla L. Manual for the ASEBA School-Age Forms and Profiles. Burlington: University of Vermont, Research Center for Children, Youth, and Families; 2001.

[2] American Psychiatric Association. Diagnostic and Statistical Manual of mental disorders (5th ed.). Arlington: Author; 2013.

[3] Arnett AB, Beighley JS, Kurtz-Nelson EC, Hoekzema K, Wang T, Bernier RA, Eichler EE (2020). Developmental predictors of cognitive and adaptive outcomes in genetic subtypes of autism spectrum disorder. Autism Res.

[4] Arnett AB, Rhoads CL, Hoekzema K, Turner TN, Gerdts J, Wallace AS, Bernier RA (2018). The autism spectrum phenotype in ADNP syndrome. Autism Res.

[5] Beighley JS, Hudac CM, Arnett AB, Peterson JL, Gerdts J, Wallace AS, Bernier RA (2020). Clinical phenotypes of carriers of mutations in CHD8 or its conserved target genes. Biol Psychiatry.

[6] Bernier R, Golzio C, Xiong B, Stessman HA, Coe BP, Penn O, Eichler EE (2014). Disruptive CHD8 mutations define a subtype of autism early in development. Cell.

[7] Bernier R, Hudac CM, Chen Q, Zeng C, Wallace AS, Gerdts J, consortium SV (2017). Developmental trajectories for young children with 16p11.2 copy number variation. Am J Med Genet B Neuropsychiatr Genet.

[8] Blakeley-Smith A, Meyer AT, Boles RE, Reaven J (2021). Group cognitive behavioural treatment for anxiety in autistic adolescents with intellectual disability: a pilot and feasibility study. J Appl Res Intellect Disabil.

[9] Chen L-S, Xu L, Huang T-Y, Dhar SU (2013). Autism genetic testing: a qualitative study of awareness, attitudes, and experiences among parents of children with autism spectrum disorders. Genet Med.

[10] Costello H, Bouras N (2006). Assessment of mental health problems in people with intellectual disabilities. Isr J Psychiatry Relat Sci.

[11] Dingemans AJM, Truijen KMG, van de Ven S, Bernier R, Bongers EMHF, Bouman A, de Vries BBA (2022). The phenotypic spectrum and genotype-phenotype correlations in 106 patients with variants in major autism gene CHD8. Transl Psychiatry.

[12] Douzgou S, Liang HW, Metcalfe K, Somarathi S, Tischkowitz M, Mohamed W, Study D.D.D (2019). The clinical presentation caused by truncating CHD8 variants. Clin Genet.

[13] Earl RK, Turner TN, Mefford HC, Hudac CM, Gerdts J, Eichler EE, Bernier RA (2017). Clinical phenotype of ASD-associated. Mol Autism.

[14] Earl RK, Ward T, Gerdts J, Eichler EE, Bernier RA, Hudac CM (2021). Sleep problems in children with ASD and gene disrupting mutations. J Genet Psychol.

[15] Elliott CD (2007). Differential ability scales.

[16] Esbensen AJ, Hoffman EK, Shaffer R, Chen E, Patel L, Jacola L (2018). Reliability of parent report measures of behaviour in children with down syndrome. J Intellect Disabil Res.

[17] Fastman J, Kolevzon A (2023). ADNP syndrome: a qualitative assessment of symptoms, therapies, and challenges. Children (Basel).

[18] Fenster R, Ziegler A, Kentros C, Geltzeiler A, Green Snyder L, Brooks E, Chung WK (2022). Characterization of phenotypic range in DYRK1A haploinsufficiency syndrome using standardized behavioral measures. Am J Med Genet A.

[19] Fischbach GD, Lord C (2010). The Simons simplex collection: a resource for identification of autism genetic risk factors. Neuron.

[20] Fok M, Bal VH (2019). Differences in profiles of emotional behavioral problems across instruments in verbal versus minimally verbal children with autism spectrum disorder. Autism Res.

[21] Fu JM, Satterstrom FK, Peng M, Brand H, Collins RL, Dong S, Talkowski ME (2022). Rare coding variation provides insight into the genetic architecture and phenotypic context of autism. Nat Genet.

[22] Halvorsen MB, Helverschou SB, Axelsdottir B, Brøndbo PH, Martinussen M (2023). General measurement tools for assessing mental health problems among children and adolescents with an intellectual disability: a systematic review. J Autism Dev Disord.

[23] Hanly C, Shah H, Au PYB, Murias K (2021). Description of neurodevelopmental phenotypes associated with 10 genetic neurodevelopmental disorders: a scoping review. Clin Genet.

[24] Helland SS, Røysamb E, Wang MV, Gustavson K (2018). Language difficulties and internalizing problems: bidirectional associations from 18 months to 8 years among boys and girls. Dev Psychopathol.

[25] Helsmoortel C, Vulto-van Silfhout AT, Coe BP, Vandeweyer G, Rooms L, van den Ende J, Van der Aa N (2014). A SWI/SNF-related autism syndrome caused by de novo mutations in ADNP. Nat Genet.

[26] Hossain MM, Khan N, Sultana A, Ma P, McKyer ELJ, Ahmed HU, Purohit N (2020). Prevalence of comorbid psychiatric disorders among people with autism spectrum disorder: an umbrella review of systematic reviews and meta-analyses. Psychiatry Res.

[27] Hudac CM, Friedman NR, Ward VR, Estreicher RE, Dorsey GC, Bernier RA, Neuhaus E. Characterizing Sensory Phenotypes of Subgroups with a Known Genetic Etiology Pertaining to Diagnoses of Autism Spectrum Disorder and Intellectual Disability. J Autism Dev Disord 2023;1-16 10.1007/s10803-023-05897-9 10.1007/s10803-023-05897-9 PMC10083138 37031308

[28] Iossifov I, O'Roak BJ, Sanders SJ, Ronemus M, Krumm N, Levy D, Wigler M (2014). The contribution of de novo coding mutations to autism spectrum disorder. Nature.

[29] Iossifov I, Ronemus M, Levy D, Wang Z, Hakker I, Rosenbaum J, Wigler M (2012). De novo gene disruptions in children on the autistic spectrum. Neuron.

[30] Jeste SS, Geschwind DH (2014). Disentangling the heterogeneity of autism spectrum disorder through genetic findings. Nat Rev Neurol.

[31] Kaufman L, Ayub M, Vincent JB (2010). The genetic basis of non-syndromic intellectual disability: a review. J Neurodev Disord.

[32] Kurtz-Nelson EC, Rea HM, Petriceks AC, Hudac CM, Wang T, Earl RK, Neuhaus E (2023). Characterizing the autism spectrum phenotype in DYRK1A-related syndrome. Autism Res.

[33] Lai MC, Kassee C, Besney R, Bonato S, Hull L, Mandy W, Ameis SH (2019). Prevalence of co-occurring mental health diagnoses in the autism population: a systematic review and meta-analysis. Lancet Psychiatry.

[34] Lai MC, Lombardo MV, Baron-Cohen S (2014). Autism. Lancet.

[35] Lord C, Rutter M, DiLavore PC, Risi S, Gotham K, Bishop S. Autism Diagnostic Observation Schedule, Second Edition (ADOS-2) Manual (Part I): Modules 1–4. Torrance: Western Psychological Services; 2012.

[36] Lord C, Rutter M, Le Couteur A (1994). Autism Diagnostic Interview - Revised: A revised version of a diagnostic interview for caregivers of individuals with possible pervasive developmental disorders. J Autism Dev Disord.

[37] Lord C, Rutter M, Le Couteur A (1994). Autism Diagnostic Interview-Revised: a revised version of a diagnostic interview for caregivers of individuals with possible pervasive developmental disorders. J Autism Dev Disord.

[38] Majnemer A, Rosenblatt B (1994). Reliability of parental recall of developmental milestones. Pediatr Neurol.

[39] McCarthy SE, Gillis J, Kramer M, Lihm J, Yoon S, Berstein Y, Corvin A (2014). De novo mutations in schizophrenia implicate chromatin remodeling and support a genetic overlap with autism and intellectual disability. Mol Psychiatry.

[40] Mitchel MW, Myers SM, Heidlebaugh AR, Taylor CM, Rea H, Neuhaus E, Eichler EE, Adam MP, Mirzaa GM, Pagon RA, Wallace SE, Bean LJH, Gripp KW, Amemiya A (2022). CHD8-Related Neurodevelopmental Disorder with Overgrowth. GeneReviews.

[41] Morison LD, Braden RO, Amor DJ, Brignell A, van Bon BWM, Morgan AT (2022). Social motivation a relative strength in DYRK1A syndrome on a background of significant speech and language impairments. Eur J Hum Genet.

[42] Mullen EM. Mullen Scales of Early Learning (AGS ed.). Circle Pines: American Guidance Service Inc ; 1995.

[43] Neo WS, Suzuki T, Kelleher BL (2021). Structural validity of the Child Behavior Checklist (CBCL) for preschoolers with neurogenetic syndromes. Res Dev Disabil.

[44] Neuhaus E, Kang VY, Kresse A, Corrigan S, Aylward E, Bernier R, Webb SJ (2022). Language and aggressive behaviors in male and female youth with autism spectrum disorder. J Autism Dev Disord.

[45] Neuhaus E, Osuna A, Tagavi DM, Shah-Hosseini S, Simmons S, Gerdts J, Thompson AD (2022). Clinical characteristics of youth with autism or developmental disability during inpatient psychiatric admission. J Clin Med.

[46] O'Roak BJ, Vives L, Fu W, Egertson JD, Stanaway IB, Phelps IG, Shendure J (2012). Multiplex targeted sequencing identifies recurrently mutated genes in autism spectrum disorders. Science.

[47] O'Roak BJ, Vives L, Girirajan S, Karakoc E, Krumm N, Coe BP, Eichler EE (2012). Sporadic autism exomes reveal a highly interconnected protein network of de novo mutations. Nature.

[48] Pedhazur EJ. Multiple regression in behavioral research : explanation and prediction (3rd ed.). Belmont: Wadsworth; 1997.

[49] Reaven J, Blakeley-Smith A, Culhane-Shelburne K, Hepburn S (2012). Group cognitive behavior therapy for children with high-functioning autism spectrum disorders and anxiety: a randomized trial. J Child Psychol Psychiatry.

[50] Reaven J, Blakeley-Smith A, Leuthe E, Moody E, Hepburn S (2012). Facing your fears in adolescence: cognitive-behavioral therapy for high-functioning autism spectrum disorders and anxiety. Autism Res Treat.

[51] Sakyi GJ, Mire SS, Goin-Kochel RP, Murali CN, Day SX. International Journal of Developmental Disabilities 2023;1–11. 10.1080/20473869.2023.2197310 10.1080/20473869.2023.2197310 PMC11774163 39882422

[52] Satterstrom FK, Kosmicki JA, Wang J, Breen MS, De Rubeis S, An JY, Buxbaum JD (2020). Large-scale exome sequencing study implicates both developmental and functional changes in the neurobiology of autism. Cell.

[53] Shillington A, Pedapati E, Hopkin R, Suhrie K (2020). Early behavioral and developmental interventions in ADNP-syndrome: a case report of SWI/SNF-related neurodevelopmental syndrome. Mol Genet Genomic Med.

[54] Sigurdsson E, Van Os J, Fombonne E (2002). Are impaired childhood motor skills a risk factor for adolescent anxiety? Results from the 1958 U.K. birth cohort and the National Child Development Study. Am J Psychiatry.

[55] Simonoff E, Pickles A, Charman T, Chandler S, Loucas T, Baird G (2008). Psychiatric disorders in children with autism spectrum disorders: prevalence, comorbidity, and associated factors in a population-derived sample. J Am Acad Child Adolesc Psychiatry.

[56] Siper PM, Layton C, Levy T, Lurie S, Benrey N, Zweifach J, Kolevzon A (2021). Sensory reactivity symptoms are a core feature of ADNP syndrome irrespective of autism diagnosis. Genes (Basel).

[57] Stessman HA, Bernier R, Eichler EE (2014). A genotype-first approach to defining the subtypes of a complex disease. Cell.

[58] van Bon BW, Coe BP, Bernier R, Green C, Gerdts J, Witherspoon K, Eichler EE (2016). Disruptive de novo mutations of DYRK1A lead to a syndromic form of autism and ID. Mol Psychiatry.

[59] van Bon BWM, Coe BP, de Vries BBA, Eichler EE, Adam M, Mirzaa G, Pagon R, Wallace S, Bean L, Gripp K, Amemiya A (2021). DYRK1A Syndrome. GeneReviews.

[60] Van Dijck A, Vandeweyer G, Kooy R, Adam M, Mirzaa G, Pagon R, Wallace S, Bean L, Gripp K, Amemiya A (2022). ADNP-Related Disorder. GeneReviews.

[61] Van Dijck A, Vulto-van Silfhout AT, Cappuyns E, van der Werf IM, Mancini GM, Tzschach A, Consortium, A (2019). Clinical presentation of a complex neurodevelopmental disorder caused by mutations in ADNP. Biol Psychiatry.

[62] Wang T, Kim CN, Bakken TE, Gillentine MA, Henning B, Mao Y, Consortium, S (2022). Integrated gene analyses of de novo variants from 46,612 trios with autism and developmental disorders. Proc Natl Acad Sci U S A.

[63] Wechsler D (2011). Wechsler Abbreviated Scale of Intelligence - Second Edition. In.

[64] Wickstrom J, Farmer C, Green Snyder L, Mitz AR, Sanders SJ, Bishop S, Thurm A (2021). Patterns of delay in early gross motor and expressive language milestone attainment in probands with genetic conditions versus idiopathic ASD from SFARI registries. J Child Psychol Psychiatry.

[65] Zhou M, Zhang YM, Li T (2023). Knowledge, attitudes and experiences of genetic testing for autism spectrum disorders among caregivers, patients, and health providers: a systematic review. World J Psychiatry.

